# Lower versus higher dose of enteral caloric intake in adult critically ill patients: a systematic review and meta-analysis

**DOI:** 10.1186/s13054-016-1539-3

**Published:** 2016-11-04

**Authors:** Hasan M. Al-Dorzi, Abdullah Albarrak, Mazen Ferwana, Mohammad Hassan Murad, Yaseen M. Arabi

**Affiliations:** 1 College of Medicine, King Saud bin Abdulaziz University for Health Sciences, Riyadh, Saudi Arabia; 2 King Abdullah International Medical Research Center, Riyadh, Saudi Arabia; 3 Intensive Care Department, King Abdulaziz Medical City, P.O. Box 22490, Riyadh, 11426 Saudi Arabia; 4 Prince Sultan Military Medical City, Riyadh, Saudi Arabia; 5 Department of Family Medicine, King Abdulaziz Medical City, Riyadh, Saudi Arabia; 6 National & Gulf Center for Evidence Based Health Practice, Riyadh, 11426 Saudi Arabia; 7 Center for Science of Healthcare Delivery, Mayo Clinic, Rochester, MN USA; 8 Preventive Medicine, Mayo Clinic, 200 1st Street SW, Rochester, MN 55905 USA

**Keywords:** Enteral feeding, Nutrition, Intensive care unit, Cross infection, Mortality

## Abstract

**Background:**

There is conflicting evidence about the relationship between the dose of enteral caloric intake and survival in critically ill patients. The objective of this systematic review and meta-analysis is to compare the effect of lower versus higher dose of enteral caloric intake in adult critically ill patients on outcome.

**Methods:**

We reviewed MEDLINE, EMBASE, Cochrane Central Register of Controlled Trials, Cochrane Database of Systematic Reviews, and Scopus from inception through November 2015. We included randomized and quasi-randomized studies in which there was a significant difference in the caloric intake in adult critically ill patients, including trials in which caloric restriction was the primary intervention (caloric restriction trials) and those with other interventions (non-caloric restriction trials). Two reviewers independently extracted data on study characteristics, caloric intake, and outcomes with hospital mortality being the primary outcome.

**Results:**

Twenty-one trials mostly with moderate bias risk were included (2365 patients in the lower caloric intake group and 2352 patients in the higher caloric group). Lower compared with higher caloric intake was not associated with difference in hospital mortality (risk ratio (RR) 0.953; 95 % confidence interval (CI) 0.838–1.083), ICU mortality (RR 0.885; 95 % CI 0.751–1.042), total nosocomial infections (RR 0.982; 95 % CI 0.878–1.077), mechanical ventilation duration, or length of ICU or hospital stay. Blood stream infections (11 trials; RR 0.718; 95 % CI 0.519–0.994) and incident renal replacement therapy (five trials; RR 0.711; 95 % CI 0.545–0.928) were lower with lower caloric intake. The associations between lower compared with higher caloric intake and primary and secondary outcomes, including pneumonia, were not different between caloric restriction and non-caloric restriction trials, except for the hospital﻿ stay which was lo﻿nger with lower caloric intake in the c﻿aloric restriction trials.

**Conclusions:**

We found no association between the dose of caloric intake in adult critically ill patients and hospital mortality. Lower caloric intake was associated with lower risk of blood stream infections and incident renal replacement therapy (five trials only). The heterogeneity in the design, feeding route and timing and caloric dose among the included trials could limit our interpretation. Further studies are needed to clarify our findings.

**Electronic supplementary material:**

The online version of this article (doi:10.1186/s13054-016-1539-3) contains supplementary material, which is available to authorized users.

## Background

Nutritional support is essential in the management of adult critically ill patients [1, 2]. Supported by randomized controlled trials (RCTs) and systematic reviews [2], early initiation of enteral nutrition (EN) has been shown to be associated with better outcome compared to late EN. However, it is less clear what the most appropriate caloric dose is. Based on expert opinion, observational studies and small RCTs [3–7], it has been generally recommended to provide full caloric requirement to critically ill patients [8]. To achieve this goal, interventions to augment caloric intake, such as the implementation of protocols [9, 10], prokineteic agents and postpyloric tube placement [11] have been proposed, even though studies have not demonstrated improvement in clinical outcomes. On the contrary, several observational studies observed better outcome with lower enteral caloric intake [12, 13].

These conflicting results may be related to differences in study populations, selection bias and immortal time bias (nutritional intake is better for patients who survive and have a longer ICU stay). Prescribed hypocaloric nutrition has recently been tested in several randomized controlled studies [14–16]. Although parenteral nutrition (PN) differs from EN in indications, physiologic effects and complications, studies have shown that lower caloric intake with PN was associated with better clinical outcomes [17]. As such the clinical practice guidelines of the Society of Critical Care Medicine and American Society for Parenteral and Enteral Nutrition recommended that mild permissive underfeeding should be considered in critically ill patients receiving PN, at least in certain high-risk groups [8, 18]. On the other hand, the evidence on the relationship between enteral caloric intake and survival remains conflicting and has ignited heated discussion in the critical care literature [19, 20].

Given the present controversies, a systematic review that includes RCTs is likely to produce more reliable effect estimates. Therefore, we conducted this systematic review and meta-analysis to compare the impact of lower versus higher dose of enteral caloric intake in adult critically ill patients on mortality and other important outcomes.

## Methods

This systematic review is reported according to the statement of Preferred Reporting Items for Systematic Reviews and Meta-analyses (PRISMA) [21].

### Literature search

We conducted a comprehensive search of several databases from the earliest inception of each database to November 2015 for randomized trials examining the effect of EN dose on the outcomes of critically ill patients. The databases included Ovid Medline In-Process and Other Non-Indexed Citations, Ovid MEDLINE, Ovid EMBASE, Ovid Cochrane Central Register of Controlled Trials, Ovid Cochrane Database of Systematic Reviews, and Scopus. The search strategy was designed and conducted by an experienced librarian. The detailed search strategy including that of Scopus is presented in Additional file 1.

We also conducted a manual search of the bibliographies of all selected articles, systematic reviews on nutritional support in critically ill patients and studies published as abstracts in the preceding five meetings of the American Thoracic Society (2010–2015), Society of Critical Care Medicine (2010–2015), American College of Chest Physicians (2010–2015), American Society for Parenteral and Enteral Nutrition (2010–2015) and European Society for Parenteral and Enteral Nutrition (2010–2015).

### Study selection

Two authors (YA and HD) independently assessed the titles and abstracts from the search results for eligibility. We included studies that met the following criteria: (1) randomized or quasi-randomized design; (2) enrolled adults who were critically ill and required care in an ICU setting; (3) primarily compared two doses of EN; (4) reported caloric intake either in absolute values (i.e., in kcal) or in percentage of caloric requirement as defined by authors; and (5) had a meaningful difference in caloric intake between the two groups (statistically significant or if the difference was ≥10 %). The 10 % difference was defined a priori by the authors of this review as the minimally significant difference.

We excluded studies: (1) of PN as a primary intervention; (2) that compared early versus late EN; (3) that assessed enteral formulae that had immune-modulating ingredients; (4) that evaluated postpyloric placement of the feeding tube for gastrointestinal reasons (such as pancreatitis) and not primarily to increase caloric intake; (5) that were cluster randomized or crossover studies; (6) that were trials with only surrogate outcomes (such as nutritional, biochemical, economic or quality of life assessment endpoints); and (7) that were trials published in abstract form only or in a non-English language.

The included trials in this systematic review fell in one of two categories. The first category was studies that had caloric restriction as the primary intervention defined as deliberate reduction of caloric intake from the estimated requirement. In these studies, which we refer to in the rest of the paper as caloric restriction trials, the lower dose included trophic feeding (minimal amounts of calories, i.e., 20 kcal/h) [15, 16] and hypocaloric feeding or permissive underfeeding (moderate amount of calories, i.e., close to 50 % of caloric requirement) [14, 22, 23]. In these trials, the higher dose was standard or eucaloric feeding (usually 70–100 % of caloric need) [14–16, 22, 23]. The second category, which we referred to as non-caloric restriction trials, included studies in which an intervention was tested that led directly or indirectly to change in caloric intake. These interventions included, but were not limited to, protocol implementation, postpyloric tube placement and the use of prokinetics. We resolved differences by discussion. All potentially eligible studies were retrieved in full.

### Risk of bias assessment

Two reviewers (HD and YA) independently assessed the methodological quality of each trial using the Cochrane Collaboration tool for Assessing Risk of Bias [24]. Any discrepancies between the two reviewers were resolved through discussion. The measure of agreement between reviewers was calculated using kappa statistics.

### Data extraction

Two non-blinded reviewers (HD and AB) independently abstracted pertinent data from the trials using a standardized predefined form. Extracted data included study design, study size, study setting, patient population, reported illness severity score, interventions and their duration and caloric intake (mean and percentage of estimated caloric target) in each arm. The primary outcome of this review was all-cause hospital mortality. Other important outcomes were chosen a priori and included ICU mortality, bloodstream infection as defined by the authors, pneumonia as defined by the authors, all infections, incident or new renal replacement therapy, mechanical ventilation duration and ICU and hospital length of stay (LOS). For published reports with insufficient information, we attempted to contact the corresponding author for clarification. Two authors replied and the two studies were later excluded.

### Statistical analysis

From each study, we extracted the number of events and sample size in each arm (binary outcomes) and the mean with a measure of variability (continuous outcomes). Meta-analysis was performed using the random-effects model as described by DerSimonian and Laird [25]. The pooled-effect estimates of lower versus higher caloric intake were reported as risk ratio (RR) and weighted mean difference (WMD) with 95 % confidence intervals (CIs).

We performed subgroup analyses with stratification by caloric restriction versus non-caloric-restriction trials which had mean patients’ age <65 versus those with mean age ≥65 years, trials which had mean Acute Physiology and Chronic Health Evaluation (APACHE) II score <20  versus those with mean APACHE II ≥20, trials in which the lower calorie group received <60 %  versus those in which the lower calorie group received ≥60 % of requirement and trials in which﻿ the calorie difference between the two groups <20%  versus those with the difference ≥20 %. We tested for interaction between the subgroups and considered the interaction test to be significant when its *p* value was <0.05. We also conducted meta-regression to assess the effect of the difference in caloric intake between the arms in each study and of the amount of provided calories in the lower calorie group in each study on hospital mortality.

All statistical tests were two-sided and a *p* value <0.05 was considered statistically significant. The degree of heterogeneity among the studies was assessed by the *I* ^2^ statistic [26]. Between-study heterogeneity was considered low if *I* ^2^ was <30 % whereas *I* ^2^ >50 % represented substantial heterogeneity. A small study bias or publication bias was assessed by visual inspection of the funnel plot and by conducting the Egger regression test. We used the Comprehensive Meta analysis Software (Version 3, Englewood, NJ, USA) for all analyses.

## Results

**Fig. 1:**
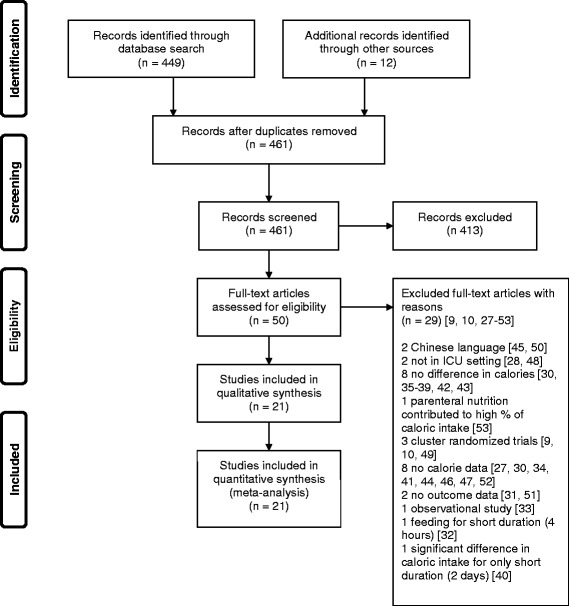
Preferred Reporting Items for Systematic Reviews and Meta-analyses (*PRISMA*) flow diagram

Figure 1 shows the flow chart of the study selection process. Through the electronic database search, we identified 449 citations. We excluded 413 studies after screening the titles and abstracts. The manual search of abstracts from conferences and of bibliographies yielded 12 additional studies that met the inclusion criteria. The full texts of 50 studies were evaluated and 29 studies were excluded [9, 10, 27–53] for different reasons as shown in Fig. 1. Twenty-one studies were eligible for data extraction and analysis [14–16, 22, 23, 54–69]. We followed the intention-to-treat principle whenever possible.

### Description of included studies

A summary of the included studies is presented in Table 1. The total sample size was 4717 patients (2365 in the lower caloric intake group and 2352 in the high caloric group). Seven studies [14–16, 22, 23, 68, 69] directly compared caloric restriction provided by enteral tube feeding with standard feeding and 14 assessed the effect of an intervention that led to a significant difference in caloric intake. The mean difference in caloric intake was 445 kcal (range 165–1118 kcal). The difference in calories between the intervention groups corresponded to 14–78.8 % of the higher caloric intake.

**Table 1:** Characteristics of studies included in the systematic review

Author, year	Population	Design	Number of patients	Age (mean)	Male (%)	APACHE II (mean)	MV	Intervention (daily caloric intake)	Control (daily caloric intake)	Duration of intervention
Montecalvo et al., 1992 [54]^a^	Medical and surgical	RCT	38	50.5 years in the intervention group, 44.8 years in the control group	60.5	Acute physiology score of APACHE II 24.0 in the intervention group, 21.7 in the control group	Not reported Probably all	Jejunal feeding (1182 ± 603 kcal; 46.9 ± 25.9 % of goal)	Gastric feeding (1466 ± 398 kcal; 61.0 ± 17 % of goal)	As long as tube feeding was required Mean tube feeding was 10 days
Kearns et al., 2000 [55]	Medical	RCT	44	54 years in the intervention group, 49 years in the control group	68	22 in the intervention group, 20 in the control group	100 %	Small intestinal feeding (1157 ± 86 kcal; 18 ± 1 kcal/kg/day; 69 ± 7 % of caloric requirement) Protein intake: 0.7 ± 0.1 g/kg/kg/day	Gastric feeding (812 ± 122 kcal; 12 ± 2 kcal/kg/day; 47 ± 7 % of caloric requirement). Protein intake 0.4 ± 0.1 g/kg/kg/day	7–10 days
Chen et al., 2006 [56]^a^	Medical	RCT	107	Similar age distribution in the control and intervention groups	76.6	Similar APACHE II distribution in both groups	100 %	Continuous feeding, with calories significantly different from the other group	Intermittent feeding	Not clear At least 7 days
Nguyen et al., 2007 [57]^a^	Medical	RCT	75	50.9 years in the intervention group, 52 years in the control group	70	23 in the intervention group, 22.6 in the control group	100 %	Combination prokinetic therapy (erythromycin and metoclopramide) Significantly higher caloric intake	Erythromycin alone Lower caloric intake	7 days
Desachy et al., 2008 [58]^a^	Medical and surgical	RCT	100	64 years in the interventional group, 58 years in the control group	69	APACHE II is not reported SAPS II: 40 in gradual feeding group, 42 in immediate feeding group	100 %	Gradual feeding 76 % of optimal calorie intake (1297 ± 331 kcal)	Immediate feeding 95 % of optimal calorie intake (1715 ± 331 kcal)	120 ± 48 hours (similar in the two groups); 45 patients were followed for maximum of 7 days
Hsu et al., 2009 [59]	Medical	RCT	121	70 years in the intervention, 67.9 years in the control group	70.2	20.5 in the intervention group, 20.3 in the control group	100 %	Nasoduodenal feeding group (1658 ± 118 kcal; 27.1 ± 7.6 kcal/kg/day) Protein intake: 1.11 ± 0.31 g/kg/day	Nasogastric feeding group (1426 ± 110 kcal; 23.5 ± 8.8 kcal/kg/day) Protein intake 0.97 ± 0.39 g/kg/day	Mean study period was 11 days
White et al., 2009 [60]	Medical	RCT	104	50 years in the intervention group, 54 years in the control group	50	30 in the intervention group, 24.5 in the control group	100 %	Postpyloric feeding (1296 kcal; 88.5 % of caloric requirement) Average protein deficit 6.5 g/day	Gastric feeding (1515 kcal; 95 % of caloric requirement) Average protein deficit 3.5 g/day	Not reported Mean gastric feeding duration was 3.1 days, mean postpyloric feeding duration was 4.0 days
Montejo et al., 2010 [61]^a^	Medical and surgical	RCT	329	65 years in the intervention group, 60 years in the control group	65	19.4 in the intervention group, 18.9 in the control group	100 %	High gastric residual volume of 500 ml (diet volume ratio in the first week = 88.2 %)	Low gastric residual volume of 200 ml (diet volume ratio in the first week = 84.5 %)	The duration of enteral nutrition (maximum of 28 days)
Acosta-Escribano et al., 2010 [62]^a^	Severe traumatic brain injury	RCT	104	35 years in the intervention group, 41 years in the control group	86.5	16 in the intervention group, 18 in the control group	100 %	Transpyloric feeding (92 % of the feeding volume given)	Gastric feeding (84 % of the feeding volume given)	Not reported
Arabi et al., 2011 [14]	Medical and surgical	RCT	240	50.3 years in the intervention group, 5.19 years in the control group	68.3	25.2 in the intervention group, 25.3 in the control group	99.2 %	Permissive under feeding: 59 % of requirement (1066 ± 306 kcal; 13.9 kcal/kg/day) Protein intake 0.61 g/kg/day	Target feeding 71.4 % of requirement (1251 ± 432 kcal; 16.4 kcal/kg/day) Protein intake 0.57 g/kg/day	Duration of enteral feeding or ICU discharge
Singer et al., 2011 [63]	Medical, surgical and trauma	RCT	130	59 years in the intervention group, 62 years in the control group	58	22.1 in the intervention group, 22.4 in the control group	100 %	Tight caloric intake according to indirect calorimetry (2086 ± 467 kcal) Protein intake 0.95 g/kg/day	Standard caloric intake at 25 kcal/kg/day (1480 ± 356 kcal) Protein intake 0.68 g/kg/day	Not clear. Till day 14 or discharge from the ICU
Rice et al., 2011 [15]	Acute respiratory failure	RCT	200	53 years in the intervention group, 54 years in the control group	44	26.9 in both groups	100 %	Trophic feeding (300 ± 149 kcal; 15.8 ± 11 % of caloric requirement) Protein intake: 0.13 g/kg/day	Full feeding (1418 ± 686 kcal; 74.8 ± 38.5 % of caloric requirement) Protein intake 0.66 g/kg/day	6 days
Rice et al., 2012 [16]^a^	ARDS patients (medical, surgical and trauma)	RCT	1000	52 years in the intervention and control groups	51	APACHE III: 92 in the intervention group, 90 in the control group	100 %	Trophic feeding (400 kcal; 25 % of estimated non-protein caloric requirement)	Full feeding (1300 kcal; 80 % of estimated caloric requirement)	6 days
Huang et al., 2012 [64]	Medical	RCT	101	70.9 years in the intervention group, 68.3 years in the control group	71	21.0 in the intervention group, 19.6 in the control group	100 %	Nasoduodenal feeding (1575 kcal; 90.4 % of target energy intake). Protein intake 93.2 ± 26.9 % of target	Nasogastric feeding (1343 kcal; 76.2 % of target energy intake) Protein intake 78.6 ± 28.5 % of target	21 days
Reignier et al., 2013 [65]^a^	Medical and surgical	RCT	449	61 years in the intervention group, 62 years in the control group	70	Baseline SOFA 8 for both groups	100 %	Not monitoring residual gastric volume (calorie deficit 319 kcal)	Monitoring residual gastric volume (calorie deficit 509 kcal)	Not clear Follow up for 90 days
Rugeles et al., 2013 [23]	Medical and surgical	RCT	80	53.3 years in the intervention group, 55.7 years in the control group	58	13.9 in the intervention group, 15.1 in the control group		Hyperproteic hypocaloric enteral nutrition as 15 kcal/kg/day (756 kcal) Protein intake 1.4 g/kg/day	Standard nutritional regimen as 25 kcal/kg/day (921 kcal) Protein intake 0.76 g/kg/day	7 days
Peake et al., 2014 [66]	Mechanically ventilated (medical and surgical)	RCT	112	56.4 years in the intervention group, 56.5 years in the control group	74	23 in the intervention group, 22 in the control group	100 %	Nutritional formula 1.5 kcal/ml (1832 ± 381 kcal; 27.3 ± 7.4 kcal/kg; 96.0 % of requirement) Protein intake 75 % of target	Nutritional formula 1 kcal/ml (1259 ± 428 kcal; 19.0 ± 6.0 kcal/kg; 68.4 % of requirement) Protein intake 79 % of target	10 days
Charles et al., 2014 [22]	Surgical/ trauma	RCT	83	50.4 years in the intervention group, 53.4 years in the control group	71	16.6 in the intervention group, 17.3 in the control group	62.7 %	Hypocaloric feeding: 50 % of estimated requirement as 12.5-15 kcal/kg/day (982 ± 61 kcal; 12.3 ± 0.7 kcal/kg/day) Protein intake 1.1 g/kg/day	Eucaloric feeding 100 % of estimated requirement as 25–30 kcal/kg/day (1338 ± 92 kcal; 17.1 ± 1.1 kcal/kg/day) Protein intake 1.1 g/kg/day	Not clear
Braunschweig et al., 2015 [67]	Acute lung injury patients (medical and surgical)	RCT	78	52.5 years in the intervention group, 58.6 years in the intervention group	51.2	23.4 in the intervention group, 27.7 in the control group		Intensive medical nutrition: >75 % of estimated energy and protein needs by a multifaceted approach (1798 ± 509 kcal; 25.4 ± 6.6 kcal/kg/day; 84.7 % of energy needs) Protein intake 0.95 g/kg/day	Standard nutrition support care (1221 ± 423 kcal; 16.6 ± 5.6 kcal/kg/day; 55.4 % of energy needs) Protein intake 0.68 g/kg/day	Till hospital discharge
Arabi et al., 2015 [68]	Medical and surgical	RCT	894	50.2 years in the intervention group, 50.9 years in the control group	64.2	21.0 in the intervention and control groups	96.8 %	Permissive underfeeding 40–60 % of caloric requirements: (835 ± 297 kcal; 46 % of requirement) Protein intake 0.72 g/kg/day	Standard feeding: 70–100 % of caloric requirements (1299 ± 467 kcal; 71 % of requirement) Protein intake 0.73 g/kg/day	Up to 14 days
Doig et al., 2015 [69]^a^	Medical and surgical	RCT	331	59 years in the intervention group, 61 years in the control group	58.6	18 in the intervention and control groups	91 %	Protocolized caloric restriction 20 kcal/h for ≥2 days then caloric intake adjusted depending on serum phosphate	Standard care, mean caloric intake at enrolment 68.5 kcal/h	At least 4 days

^a^Protein intake not reported. *APACHE* Acute Physiology and Chronic Health Evaluation, *ARDS* acute respiratory distress syndrome, *MV* mechanical ventilation, *RCT* randomized controlled trial, *SAPS* Simplified Acute Physiology Score. *SOFA* Sequential Organ Failure Assessment

Table 2 describes the risk of bias assessment of the studies and shows that most studies had bias risk in at least one domain of the Cochrane Collaboration tool. All except two studies [57, 66] were un-blinded. Agreement between reviewers (kappa) on the tool elements was 0.774 (95 % CI 0.651–0.892; *p* < 0.001).

**Table 2:** Quality of included randomized controlled trials using the Cochrane Collaboration tool for assessing risk of bias

	Sequence generation	Concealment	Blinding	Incomplete outcome data	Selective outcome reporting	Other sources of bias	Study center	Percentage of patients lost to follow up	Source of study funding
Montecalvo et al., 1992 [54]	Computer generated	Not described	No	No	No	No	Multiple ICUs, two centers	0	Not reported
Kearns et al., 2000 [55]	Computer generated	Sealed envelope	No	No	No	No	Single	0	Ross Laboratories and the California Institute for Medical Research (partly)
Chen et al., 2006 [56]	Not described	Not described	No	No	No	No	Two ICUs, one center	0	National Science Council
Nguyen et al., 2007 [57]	Computer generated	Yes	Yes	No	No	No	Single	0	National Health and Medical Research Council (NHMRC) of Australia (partly)
Desachy et al., 2008 [58]	Not described	Not described	No	No	No	No	Two ICUs	0	Not reported
Hsu et al., 2009 [59]	Computer generated	Yes	No	No	No	No	Single	0	Kaohsiung Veterans General Hospital
White et al., 2009 [60]	Computer generated	Yes (sealed opaque envelope)	No	Yes	No	No	Single	0	Not reported
Montejo et al., 2010 [61]	Not described	Not described	No	No	No	No	Single	0	Not reported
Acosta-Escribano et al., 2010 [62]	Central randomization	Yes	No	Yes	No	No	Multicenter	0	Novartis Consumer Health (Spain)
Arabi et al., 2011 [14]	Computer generated	Yes	No	No	No	No	Single	0	King Abdulaziz City for Science and Technology
Singer et al., 2011 [63]	Computer generated	Yes	No	No	No	No	Single	0	Not reported
Rice et al., 2011 [15]	Not described	Yes (sealed opaque envelope)	No	No	No	No	Two ICUs, single center	0	National Institutes of Health (partly)
Rice et al., 2012 [16]	Web-based system	Sealed envelope	No	No	No	No	Multicenter	0	National Heart, Lung, and Blood Institute
Huang et al., 2012 [64]	Software-generated randomization	Not described	No	Yes Hospital mortality data were missing for some patients	No	No	Single	Hospital mortality data missing for 4/101 patients (4 %)	Kaohsiung Veterans General Hospital
Reignier et al., 2013 [65]	Computer-generated, interactive, web-response system	Yes	No	No	No	No	Multicenter	0	The Centre Hospitalier Departemental de la Vendee
Rugeles et al., 2013 [23]	Computer-generated random allocations	No Sealed envelopes were used but one investigator knew patient allocation	No One investigator knew patient allocation.	Yes Mortality was reported as one of the secondary endpoints but was not reported	Yes	Yes Many patients were excluded from analysis with uncertainty if exclusion criteria were determined a priori.	Single	0	Lafrancol S.A
Peake et al., 2014 [66]	Web-based system	Centralized, web-based randomization schedule	Yes	No	No	No	Multicenter	One patient in the intervention group was withdrawn and one patient in the control group was lost to follow up by day 90.	The Royal Adelaide Hospital and the Australian, New Zealand College of Anaesthetists and Fresenius Kabi
Charles et al., 2014 [22]	Random number sequence	Opaque security envelopes	No	No	No	Yes The trial was stopped before achieving the target sample size because of slow enrolment	Single	0	The NIH
Braunschweig et al., 2015 [67]	Computer-generated random allocations	Sealed envelopes	No	No	No	Yes The trial was stopped before achieving the target sample size because of higher mortality, a secondary outcome, in the intervention group	Single	0	The NIH/NHLBI
Arabi et al., 2015 [68]	Computer-generated random allocations (blocks of variable size	Opaque sealed envelopes	No	No	No	No	Multicenter	9 patients lost to follow up, 3 in the intervention group and 3 in the control group	King Abdullah International Medical Research Center
Doig et al., 2015 [69]	Computer-generated random allocations (blocks of variable size)	Secure central randomization web server	No	No	No	No	Multicenter	4 patients lost to follow up (90-day interview), 2 in each group.	National Health and Medical Research Council of Australia

### Effect on mortality

**Fig. 2:**
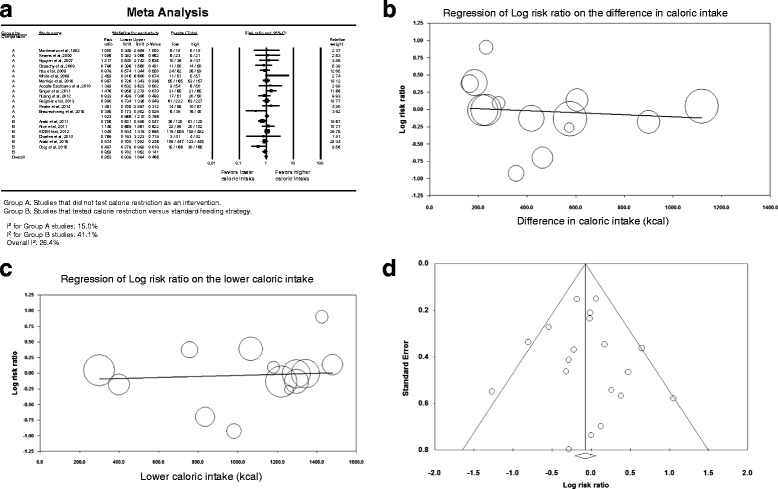
Hospital mortality. **a** Pooled risk ratio with 95 % confidence interval (*CI*) for hospital mortality, association with lower versus higher dose of enteral feeding. The random effects model was used. **b** Meta-regression for the effect of the difference in calories between the lower and higher caloric intake groups in each trial on hospital mortality. **c** Meta-regression for the effect of the caloric dose in the lower caloric intake group in each trial on hospital mortality. **d** Funnel plot for the corresponding studies

Nineteen studies provided data on hospital mortality [14–16, 22, 54, 55, 57–69]. Figure 2a describes the corresponding forest plot. Lower compared with higher caloric intake was not associated with difference in hospital mortality; the overall RR was 0.953 (95 % CI, 0.838–1.083). The association between lower compared with higher caloric intake and hospital mortality was not different between caloric restriction [14–16, 22, 68, 69] and non-caloric restriction trials [54, 55, 57–67] (interaction test *p* = 0.19).

Similarly, the association between lower compared with higher caloric intake and hospital mortality was not different between studies in which the mean age of patients was ≥65 years [59, 64] and those in which the mean age was <65 years [14–16, 22, 54, 55, 57, 58, 60–63, 65–69] (RR 0.994; 95 % CI 0.825–1.079; interaction test *p* = 0.54), between studies in which the mean APACHE II score was <20 [22, 61, 62, 69], and those in which the mean APACHE II score was ≥20 [14, 15, 54, 55, 57, 59, 60, 63, 64, 66–68] (RR 0.977; 95 % CI 0.862–1.108; interaction test *p* = 0.73), between studies [14–16, 22, 54, 68, 69] where the lower calorie group received <60 % of requirement and studies where the lower calorie group received ≥60 % [55, 57–67] (RR 0.946; 95 % CI 0.832–1.075; interaction test *p* = 0.20) and between the studies in which the calorie difference between the two groups ≥20 % [15, 16, 22, 55, 66–69] and those with calorie difference <20 % [14, 58, 60, 62, 64] (RR 0.977; 95 % CI 0.860–1.110; interaction test *p* = 0.97).

The meta-regression analysis (Fig. 2b and c) showed that hospital mortality was not influenced by the difference in caloric intake between the lower and higher calorie groups in each trial (slope = -0.0002; *p* = 0.56) or by the lower caloric dose in each trial (slope = 0.0001; *p* = 0.69). Additionally, there was low heterogeneity among the studies (*I* ^2^ = 26.4 %) and no evidence of publication bias on inspection of the funnel plot (Fig. 2d), with *p* = 0.79 for Egger’s test.

**Fig. 3:**
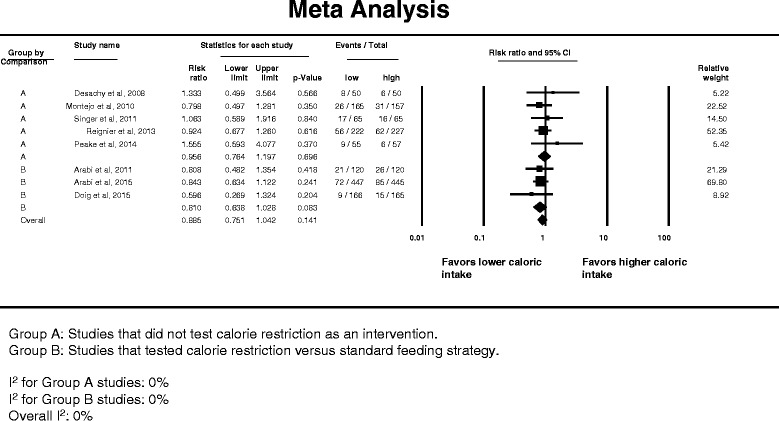
Pooled risk ratio with 95 % confidence interval (*CI*) for intensive care unit mortality associated with lower versus higher dose of enteral feeding. The random effects model was used

Eight studies had data on ICU mortality. Figure 3 presents the corresponding forest plot. The overall RR was 0.885 (95 % CI 0.751–1.042). There was no significant difference between caloric restriction and non-caloric-restriction trials (interaction test *p* = 0.32). There was no significant heterogeneity among the studies (*I* ^2^ = 0 %) and no evidence of publication bias (Egger’s test *p* = 0.40).

### Effect on infections

**Fig. 4:**
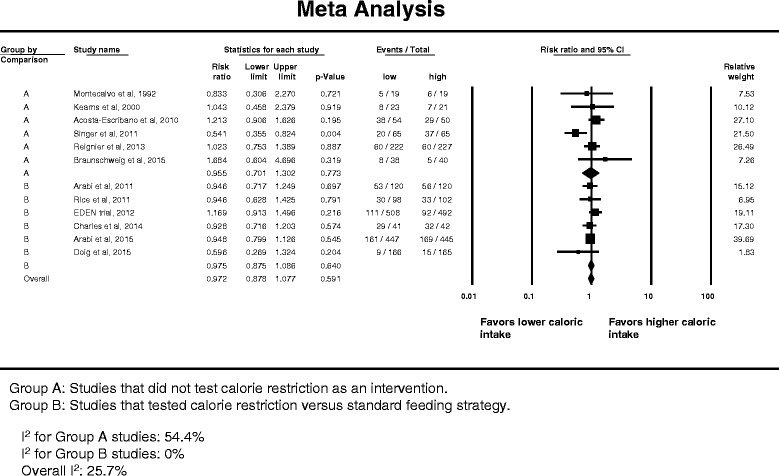
Pooled risk ratio with 95 % confidence interval (*CI*) for total infections associated with lower versus higher dose of enteral feeding. The random effects model was used

Twelve studies reported data on more than one infection [14–16, 22, 54, 55, 62, 63, 65, 67–69]. Figure 4 describes the corresponding forest plot. The overall RR was 0.972 (95 % CI, 0.878–1.077) with no significant difference between caloric restriction and non-caloric-restriction trials (interaction test *p* = 0.91). The overall heterogeneity was low (*I* ^2^ = 25.7 %).

**Fig. 5:**
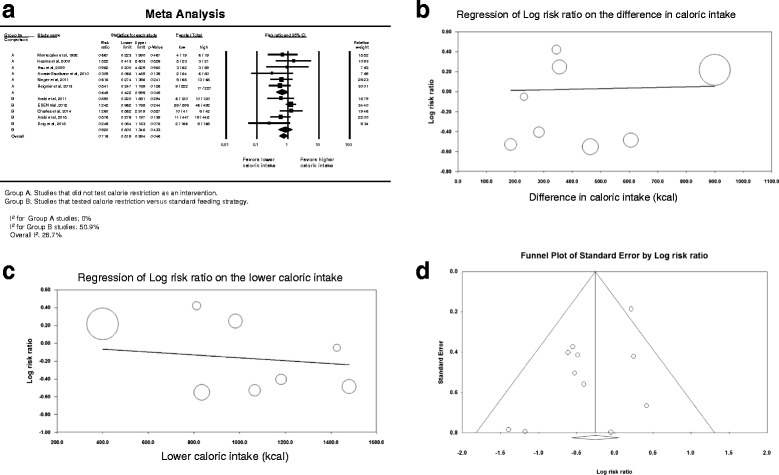
Blood stream infection. **a** Pooled risk ratio with 95 % confidence interval (*CI*) for hospital mortality associated with lower versus higher dose of enteral feeding. The random effects model was used. **b** Meta-regression for the effect of the difference in calories between the lower and higher caloric intake groups on hospital mortality. **c** Meta-regression for the effect of the caloric dose in the lower caloric intake group on hospital mortality. **d** Funnel plot for the corresponding studies

Eleven studies collected data on bloodstream infections [14, 16, 22, 54, 55, 59, 62, 63, 65, 68, 69] and showed lower risk of such infections in the lower versus higher caloric intake groups (RR 0.718; 95 % CI 0.519–0.994; Fig. 5a). The interaction test between caloric restriction and non-caloric-restriction trials was not significant (*p* = 0.48). Meta-regression showed no association between bloodstream infection risk and the amount of calorie intake in the difference in caloric intake between the intervention groups in each trial (slope = 0.0001; *p* = 0.75) and the lower calorie group in each trial (slope = -0.0002; *p* = 0.30) (Fig. 5b and c). There was low heterogeneity among the studies (*I* ^2^ = 26.7 %). The funnel plot (Fig. 5d) showed possible publication bias (Egger’s test *p* = 0.04).

**Fig. 6:**
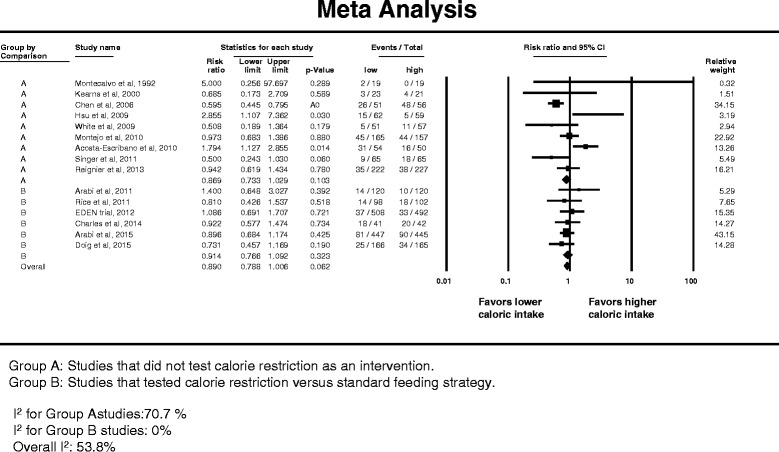
Pooled risk ratio with 95 % confidence interval (*CI*) for pneumonia associated with lower versus higher dose of enteral feeding. The random effects model was used

Fifteen studies reported data on development of pneumonia [14–16, 22, 54–56, 59–63, 65, 68, 69]. Figure 6 presents the related forest plot. The overall RR was 0.920 (95 % CI 0.784–1.080) with no significant difference between caloric restriction and non-caloric-restriction trials (interaction test *p* = 0.88). There was substantial heterogeneity among the studies (*I* ^2^ = 53.8 %).

### Effect on incident renal replacement therapy

**Fig. 7:**
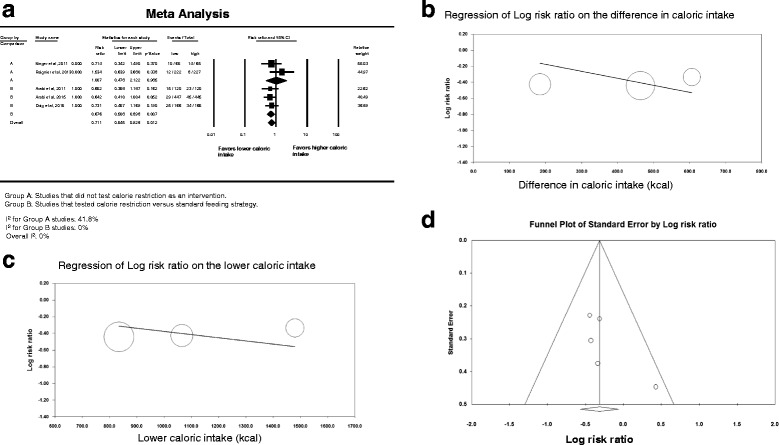
Incident renal replacement therapy. **a** Pooled risk ratio with 95 % confidence interval (*CI*) for incident renal replacement therapy, association with lower versus higher dose of enteral feeding. The random effects model was used. **b** Meta-regression for the effect of the difference in calories between the lower and higher caloric intake groups in each trial on incident renal replacement therapy. **c** Meta-regression for the effect of the caloric dose in the lower caloric intake group in each trial on incident renal replacement therapy. **d** Funnel plot for the corresponding studies

Five studies reported data on incident renal replacement therapy [14, 63, 65, 68, 69]. The overall RR was 0.711 (95 % CI 0.545–0.928) (Fig. 7a) with no significant difference between caloric restriction and non-caloric-restriction trials (test of interaction *p* = 0.33). The meta-regression (Fig. 7b and c) showed significant association between incident renal replacement therapy and the difference in caloric intake between the lower and higher calorie groups in each study (slope = -0.0009; *p* = 0.02) and the lower caloric dose in each study (slope = -0.0004; *p* = 0.02). There was low heterogeneity (*I* ^2^ = 0 %) with the funnel plot shown in Fig. 7d (Egger’s test *p* = 0.17).

### Effect on length of stay

**Fig. 8:**
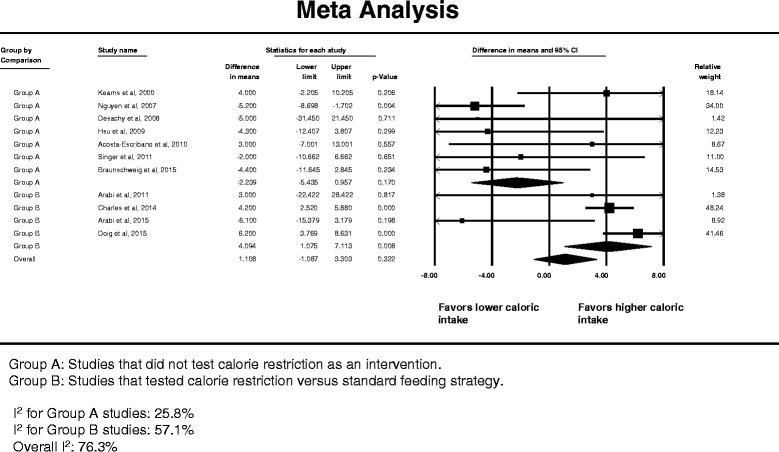
Pooled risk ratio with 95 % confidence interval (*CI*) for hospital length of stay associated with lower versus higher dose of enteral feeding. The random effects model was used

Eleven studies provided data (means with standard deviations) on hospital LOS [14, 22, 55, 57–60, 62, 67–69]. The pooled analysis showed no difference in hospital stay (WMD = +1.11 days; 95 % CI -1.09–3.30 days) (Fig. 8). However, the association of lower versus higher caloric intake and hospital LOS was different between caloric restriction and non-caloric-restriction trials (interaction test *p* = 0.005). In the caloric restriction trials, the hospital LOS was longer with lower compared with higher caloric intake (WMD = +4.09 days; 95 % CI 1.08–7.11 days), whereas it was not different between lower and higher dose caloric intake in the non-caloric restriction trials (Fig. 8). There was substantial heterogeneity among the studies (*I* ^2^ = 76.3 %). However, there was no evidence of publication bias (Egger’s test *p* = 0.11). Meta-regression analysis showed that the hospital LOS was not influenced by the difference in caloric intake between the lower and higher calorie groups in each trial or by the lower calorie dose in each trial.

**Fig. 9:**
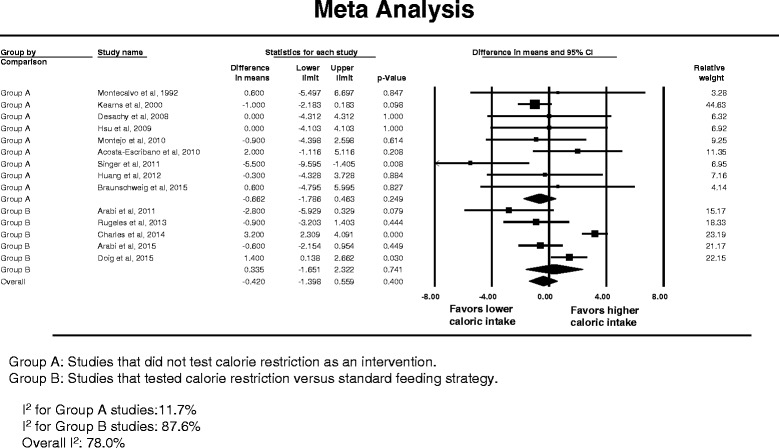
Pooled risk ratio with 95 % confidence interval (*CI*) for intensive care unit length of stay associated with lower versus higher dose of enteral feeding. The random effects model was used

Fourteen studies provided data on ICU LOS [14, 22, 23, 54, 55, 58, 59, 61–64, 67–69] with no significant difference between the lower and higher caloric intake groups (WMD = -0.13 days (95 % CI -1.45–1.19 days) (Fig. 9). The association between lower compared with higher caloric intake and ICU LOS was not different between caloric restriction and non-caloric-restriction trials (test of interaction *p* = 0.39). There was a substantial heterogeneity among the studies (*I* ^2^ = 78.0 %). Egger’s test *p* was 0.07.

**Fig. 10:**
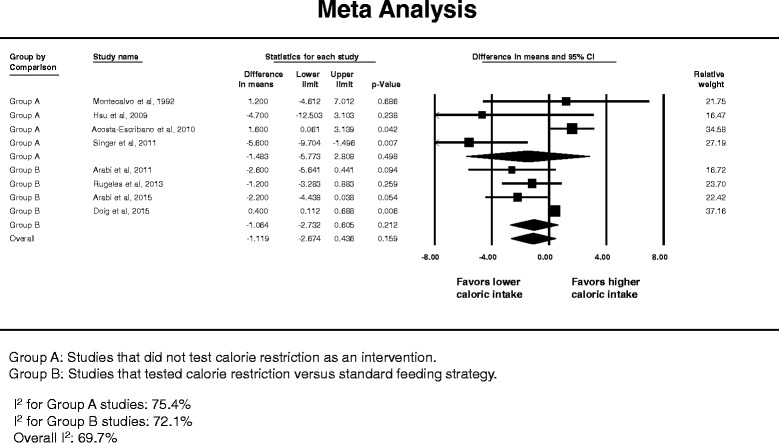
Pooled risk ratio with 95 % confidence interval (*CI*) for the duration of mechanical ventilation associated with lower versus higher dose of enteral feeding. The random effects model was used

Eight studies had data on duration of mechanical ventilation [14, 23, 54, 59, 62, 63, 68, 69]. The meta-analysis found no difference (Fig. 10) with WMD = -1.12 days (95 % CI -2.67– 0.44 days). There was substantial heterogeneity among the studies (*I* ^2^ = 69.7 %). The association between lower compared with higher caloric intake and mechanical ventilation duration was not different between caloric restriction and non-caloric-restriction trials (test of interaction *p* = 0.89). The Egger’s test *p* value was 0.09, suggesting insignificant publication bias.

## Discussion

In this systematic review and meta-analysis of RCTs of critically ill patients in which there was a significant difference in the caloric intake via EN, we found that lower versus higher dose of caloric intake was not associated with mortality. However, patients in the lower caloric intake group had lower risk of blood stream infections and incident renal replacement therapy. The associations between lower compared with higher caloric intake and all studied outcomes were not different between caloric restriction and non-caloric restriction trials, except for the hospital LOS. Our certainty in the meta-analytic estimates is reduced due to the moderate risk of bias.

The optimal EN dose during critical illness is unknown. Malnourishment is generally associated with poor outcomes leading to the notion that replacement of full energy needs is intuitively needed. However, in critically ill patients, multiple metabolic changes may occur and differ from one patient to another. The metabolic rate is highly variable, being increased in about 50 % of patients but low in others [70]. Glycolysis is increased as the energy source is altered from predominantly fat to glucose oxidation [71]. Proteolysis is accelerated especially as prolonged starvation is associated with dysregulated metabolism, such that major stimulation of both ketogenesis and concomitant gluconeogenesis do not occur [72]. The normal suppression of lipolysis and proteolysis by the exogenous supply of fat or carbohydrates is blunted [71]. Additionally, excess caloric provision might be detrimental, particularly to the mitochondria by increasing oxygen radical production [73], and full EN may lead to gastrointestinal intolerance, which may increase infection risk. On the other hand, caloric restriction may enhance autophagy, which might be protective [74, 75]. These changes have led to the premise that lower caloric intake may have a protective effect.

The available studies on this topic produced mixed results. In this meta-analysis of RCTs in which critically ill patients received significantly different caloric intake, we found no difference in the primary outcome of hospital mortality. The results were similar in caloric restriction and non-caloric restriction trials and when studies were stratified based on age, severity of illness, amount of calories in the lower calorie group and the difference in calories between the intervention groups. Moreover, there was no clear dose–effect relationship as observed in the meta-regression. Marik and Hooper have recently published a systematic review and meta-analysis of six trials that assessed the outcomes of ICU patients randomized to either normocaloric or hypocaloric feeding and also found no difference in mortality and infectious complications [76]. However, our study differed in its inclusion and exclusion criteria and thus included 21 trials, which evaluated various interventions that resulted in different caloric intake. We also analyzed multiple subgroups and performed meta-regression.

We note that in one of the included trials, the TICACOS trial [63], the difference in energy intake was largely provoked by greater administration of PN rather than EN. However, we included the study because it met our a priori inclusion criterion; the mean enterally delivered energy in the study group was 1515 ± 756 kcal/day compared with 1316 ± 456 kcal/day in the control group (*p* = 0.09), which is more than 10 % [63]. We also note that in the included INTACT trial [67], the number of days between hospital admission and enrollment were 8.8 ± 8.7 and 6.4 ± 6.6 days in the intensive medical and standard nutrition groups, respectively (Table 1) and the caloric target was 30 kcal/kg in the intensive medical group. These issues raised concerns about the occurrence of refeeding syndrome, which was not studied in the trial, and of overfeeding in the intensive medical group which may have led to early ICU deaths [67, 77–79].

The relationship between nutrition and infection risk has been evaluated in different settings. A meta-analysis found that EN versus PN was associated with a significant decrease in infectious complications in critically ill patients (relative risk 0.64, 95 % CI 0.47–0.87; *p* = 0.004) [80]. Another meta-analysis found that EN within 24 hours after elective gastrointestinal surgery compared with nil-by-mouth management reduced any infection risk (relative risk 0.72, 95 % CI 0.54–0.98; *p* = 0.04) [81]. However, the impact of EN dose in critically ill patients is not well-studied. In the current study, we have observed that lower caloric intake was associated with lower nosocomial blood stream infection risk. However, there was no effect on total infections or pneumonia. This could be related to the heterogeneity of studies and publication bias.

Caloric restriction has been shown to be reno-protective in animal models of acute kidney injury [82–84]. However, a secondary analysis in the Randomized Evaluation of Normal vs. Augmented Level of Replacement Therapy trial found that the increased caloric intake was associated with lower 90-day mortality on multivariable logistic regression analysis (odds ratio per 100 kcal increment 0.95, 95 % CI 0.91–1.00; *p* = 0.06) on multivariable logistic regression analysis [85]. This was more pronounced after excluding patients who died within 96 hours of ICU admission [85]. However, the mean caloric intake during treatment was low (approximately 11 ± 9 kcal/kg/day). Our meta-analysis identified lower incident renal replacement therapy rates in the lower calorie group, but this finding is limited by the small number of studies (n = 5) that reported on this outcome. Other possible mechanisms for the difference in renal replacement therapy, which may include differences in protein intake or fluid balance between the two groups, could not be examined in our meta-analysis. Further studies are needed to clarify this association.

Multiple observational and interventional studies have identified variable associations between EN dose and ICU and hospital LOS and mechanical ventilation duration [9, 12, 86–88]. Our meta-analysis did not identify differences in mechanical ventilation duration or ICU LOS. Lower caloric intake was associated with longer hospital LOS in the subgroup of studies that compared caloric restriction with standard feeding.

This study needs to be interpreted in the light of its strengths and limitations. Potential confounders were controlled for because of the randomized design of the included trials. For example, age and severity of illness were similar in the two arms in the included trials (Table 1). A further strength is that rather than being selective and possibly biased in selecting trials, we included all studies in which a difference in enteral intake was provoked by the intervention. As such we included not only studies on caloric restriction but also studies evaluating other interventions that affect caloric intake, such as gastric residual volume management or small bowel feeding. Such relatively wide inclusion criteria allowed the inclusion of more trials. However, this strength may have provoked a possible weakness. Studies were pooled despite different design, some of them not even focused on improving outcome by reaching feeding goals or by nutrient restriction. We have addressed this limitation by our subgroup analyses and by the meta-regression. Our study focused on EN and did not include studies that had PN as the primary intervention. Hence, the study by Heidegger et al., in which ICU patients who received <60 % of their calorimetry-calculated caloric target from EN were randomized to either EN or EN plus supplemental PN [89], was not included. Although several studies showed similar outcomes with the two routes [80, 90], we chose not to include PN trials as this may further contribute to the heterogeneity. In addition, permissive underfeeding in PN has been studied in earlier systematic reviews [8, 91].

Another limitation is related to the quality of the included studies, most of which had one or more form of bias. Furthermore, the caloric intake in all included studies in the higher feeding group was generally less than the estimated caloric requirement. This is a reflection of the difficulty in achieving full targets in cohorts of ICU patients (although it certainly is achievable in many individual patients). However, this resembles what has been shown in many observational studies and in real-world practice. Given that the full enteral intake was reached in a few studies only, the benefit of this strategy remains unclear. While some studies calculated the calories from sources other than EN and PN, such as intravenous dextrose (including medications) and propofol [14, 55, 63, 66–68], this was not a consistent approach among all trials. Most included studies have used predictive equations rather than indirect calorimetry to estimate caloric intake. However, this is consistent with usual practice in most ICUs, given the limited evidence supporting either approach. Energy requirements were estimated using different predictive formulae in all but one study, which used indirect calorimetry [63]. This method has been shown to poorly correlate with indirect calorimetry [92], which is considered to be the standard. Based on recent evidence, calorimetry-guided nutrition might be superior to predictive formula-based feeding in improving patient-centered outcomes [63, 89].

The caloric dose and target varied among the included studies and what was lower caloric intake in one study was high in another. However, the meta-regression analysis suggested that the differences in calories between groups and the amount of calories in the lower caloric intake group did not influence hospital mortality. Moreover, not all studies reported on all the outcomes, thus reducing the sample size used and the ability to find small but potentially clinically significant effects. Given the reported data in the individual studies, further analysis of the impact of protein intake could not be performed. Our systemic review highlights the limited data on this important issue and calls for further studies on the impact of protein intake on the outcomes of critically ill patients. Our meta-analysis was not an individual-patient meta-analysis and did not include economic or long-term endpoints.

## Conclusions

Lower versus higher dose of caloric intake in adult critically ill patients was not associated with differences in mortality, risk of pneumonia or mechanical ventilation duration. Lower caloric intake was associated with lower risk of blood stream infections and incident renal replacement. The great heterogeneity in the design, feeding route and timing and caloric dose among the included trials could limit the interpretation of the results of this systematic review and meta-analysis. Further studies are needed to further evaluate the association between caloric intake and renal replacement therapy in critically ill patients.

## Additional file

Additional file 1: Search strategy. (DOC 56 kb)

## Abbreviations

APACHE: Acute Physiology and Chronic Health Evaluation
CI: Confidence interval
EN: Enteral nutrition
ICU: Intensive care unit
LOS: Length of stay
PN: Parenteral nutrition
PRISMA: Preferred Reporting Items for Systematic Reviews and Meta-analyses
RR: Risk ratio
RCT: Randomized controll ed trial
WMD: Weighted mean difference
