# Supplementary material for: Lower versus higher dose of enteral caloric intake in adult critically ill patients: a systematic review and meta-analysis
Source: Crit Care. 2016 Nov 4;20:358. doi: 10.1186/s13054-016-1539-3 (PMC5097427; doi:10.1186/s13054-016-1539-3)
Supplement: Additional file 1: — Search strategy. (DOC 56 kb) [file 13054_2016_1539_MOESM1_ESM.doc]

**Search Strategy**

Ovid

Database(s): Embase 1988 to 2015 Week 48, Ovid MEDLINE(R) In-Process & Other Non-Indexed Citations and Ovid MEDLINE(R) 1946 to Present, EBM Reviews - Cochrane Central Register of Controlled Trials October 2015, EBM Reviews - Cochrane Database of Systematic Reviews 2005 to November 2015
Search Strategy:

| **#** | **Searches** | **Results** |
| --- | --- | --- |
| 1 | exp Enteral Nutrition/ | 39244 |
| 2 | exp enteric feeding/ | 21120 |
| 3 | ("enteral nutrition" or "enteric nutrition" or "feeding tube*" or "enteral feeding*" or "enteric feeding*" or "force feeding*" or "tube feeding*" or "intestinal feeding*" or "intraintestinal feeding*").mp. | 56501 |
| 4 | exp Nutritional Support/ | 56911 |
| 5 | exp caloric intake/ | 84115 |
| 6 | exp Energy Intake/ | 84115 |
| 7 | exp Nutritional Status/ | 69902 |
| 8 | ("nutritional support" or "artificial feeding*" or "energy intake" or "caloric intake" or "dietary energy" or (nutrition* adj (state or status)) or "nutritional therap*").mp. | 216066 |
| 9 | or/1-8 | 278536 |
| 10 | exp critical illness/ | 43369 |
| 11 | exp Critical Care/ | 508096 |
| 12 | exp intensive care/ | 482126 |
| 13 | exp intensive care unit/ | 162217 |
| 14 | exp Intensive Care Units/ | 162217 |
| 15 | ("critical illness" or "critically ill" or "critical care" or "intensive care" or icu or icus).mp. [mp=ti, ab, hw, tn, ot, dm, mf, dv, kw, nm, kf, px, rx, ui, sh, tx, ct] | 470872 |
| 16 | or/10-15 | 782752 |
| 17 | exp Mortality/ | 1009244 |
| 18 | exp treatment outcome/ | 1950576 |
| 19 | exp infection/ | 2848534 |
| 20 | mortality.fs. | 464953 |
| 21 | exp Respiration, Artificial/ | 191198 |
| 22 | exp artificial ventilation/ | 123106 |
| 23 | exp pneumonia/ | 270004 |
| 24 | exp "Length of Stay"/ | 174191 |
| 25 | (mortality or death or outcome or infection* or infectious or ventilat* or pneumon* or (length adj2 stay) or "artificial respirat*" or "pulmonary inflamation*" or "lung inflamation*").mp. [mp=ti, ab, hw, tn, ot, dm, mf, dv, kw, nm, kf, px, rx, ui, sh, tx, ct] | 8628525 |
| 26 | or/17-25 | 9707066 |
| 27 | (underfed or underfeeding or underfeed or overfed or overfeeding or overfeed or ((enhanc* or minimum or minimal* or maximum or maximal* low or high or dose or dosage or trophic or level or lower or higher or full) adj5 (enteral or nutrition* or feeding* or energy or caloric or calorie* or diet or diets or dietary))).mp. [mp=ti, ab, hw, tn, ot, dm, mf, dv, kw, nm, kf, px, rx, ui, sh, tx, ct] | 349334 |
| 28 | 9 and 16 and 26 and 27 | 3373 |
| 29 | randomized controlled trial/ | 793426 |
| 30 | (randomized adj2 (study or studies or trial or trials)).mp. | 1408557 |
| 31 | 29 or 30 | 1408557 |
| 32 | 28 and 31 | 913 |
| 33 | limit 32 to ("all adult (19 plus years)" or "young adult (19 to 24 years)" or "adult (19 to 44 years)" or "young adult and adult (19-24 and 19-44)" or "middle age (45 to 64 years)" or "middle aged (45 plus years)" or "all aged (65 and over)" or "aged (80 and over)") [Limit not valid in Embase,CCTR,CDSR; records were retained] | 820 |
| 34 | limit 33 to (adult <18 to 64 years> or aged <65+ years>) [Limit not valid in Ovid MEDLINE(R),Ovid MEDLINE(R) In-Process,CCTR,CDSR; records were retained] | 499 |
| 35 | limit 34 to (editorial or erratum or letter or note or addresses or autobiography or bibliography or biography or dictionary or directory or interactive tutorial or interview or lectures or legislation or news or newspaper article or patient education handout or periodical index or portraits or published erratum or video-audio media or webcasts) [Limit not valid in Embase,Ovid MEDLINE(R),Ovid MEDLINE(R) In-Process,CCTR,CDSR; records were retained] | 77 |
| 36 | 34 not 35 | 422 |
| 37 | from 28 keep 3052-3373 | 322 |
| 38 | 36 or 37 | 601 |
| 39 | remove duplicates from 38 | 432 |

Scopus

1. TITLE-ABS-KEY("enteral nutrition" or "enteric nutrition" or "feeding tube*" or "enteral feeding*" or "enteric feeding*" or "force feeding*" or "tube feeding*" or "intestinal feeding*" or "intraintestinal feeding*")
2. TITLE-ABS-KEY("nutritional support" or "artificial feeding*" or "energy intake" or "caloric intake" or "dietary energy" or (nutrition* W/1 state) or (nutrition* W/1 status) or "nutritional therap*")
3. 1 or 2
4. TITLE-ABS-KEY("critical illness" or "critically ill" or "critical care" or "intensive care" or icu or icus)
5. TITLE-ABS-KEY(mortality or death or outcome or infection* or infectious or ventilat* or pneumon* or (length W/2 stay) or "artificial respirat*" or "pulmonary inflamation*" or "lung inflamation*")
6. TITLE-ABS-KEY(underfed or underfeeding or underfeed or overfed or overfeeding or overfeed or (enhanc* W/1 enteral) or (enhanc* W/1 nutrition*) or (enhanc* W/1 feeding*) or (enhanc* W/1 energy) or (enhanc* W/1 caloric) or (enhanc* W/1 calorie*) or (enhanc* W/1 diet) or (enhanc* W/1 diets) or (enhanc* W/1 dietary) or (minimum W/1 enteral) or (minimum W/1 nutrition*) or (minimum W/1 feeding*) or (minimum W/1 energy) or (minimum W/1 caloric) or (minimum W/1 calorie*) or (minimum W/1 diet) or (minimum W/1 diets) or (minimum W/1 dietary) or (minimal* W/1 enteral) or (minimal* W/1 nutrition*) or (minimal* W/1 feeding*) or (minimal* W/1 energy) or (minimal* W/1 caloric) or (minimal* W/1 calorie*) or (minimal* W/1 diet) or (minimal* W/1 diets) or (minimal* W/1 dietary) or (maximum W/1 enteral) or (maximum W/1 nutrition*) or (maximum W/1 feeding*) or (maximum W/1 energy) or (maximum W/1 caloric) or (maximum W/1 calorie*) or (maximum W/1 diet) or (maximum W/1 diets) or (maximum W/1 dietary) or (maximal* W/1 enteral) or (maximal* W/1 nutrition*) or (maximal* W/1 feeding*) or (maximal* W/1 energy) or (maximal* W/1 caloric) or (maximal* W/1 calorie*) or (maximal* W/1 diet) or (maximal* W/1 diets) or (maximal* W/1 dietary) or (low W/1 enteral) or (low W/1 nutrition*) or (low W/1 feeding*) or (low W/1 energy) or (low W/1 caloric) or (low W/1 calorie*) or (low W/1 diet) or (low W/1 diets) or (low W/1 dietary) or (high W/1 enteral) or (high W/1 nutrition*) or (high W/1 feeding*) or (high W/1 energy) or (high W/1 caloric) or (high W/1 calorie*) or (high W/1 diet) or (high W/1 diets) or (high W/1 dietary) or (dose W/1 enteral) or (dose W/1 nutrition*) or (dose W/1 feeding*) or (dose W/1 energy) or (dose W/1 caloric) or (dose W/1 calorie*) or (dose W/1 diet) or (dose W/1 diets) or (dose W/1 dietary) or (dosage W/1 enteral) or (dosage W/1 nutrition*) or (dosage W/1 feeding*) or (dosage W/1 energy) or (dosage W/1 caloric) or (dosage W/1 calorie*) or (dosage W/1 diet) or (dosage W/1 diets) or (dosage W/1 dietary) or (trophic W/1 enteral) or (trophic W/1 nutrition*) or (trophic W/1 feeding*) or (trophic W/1 energy) or (trophic W/1 caloric) or (trophic W/1 calorie*) or (trophic W/1 diet) or (trophic W/1 diets) or (trophic W/1 dietary) or (level W/1 enteral) or (level W/1 nutrition*) or (level W/1 feeding*) or (level W/1 energy) or (level W/1 caloric) or (level W/1 calorie*) or (level W/1 diet) or (level W/1 diets) or (level W/1 dietary) or (lower W/1 enteral) or (lower W/1 nutrition*) or (lower W/1 feeding*) or (lower W/1 energy) or (lower W/1 caloric) or (lower W/1 calorie*) or (lower W/1 diet) or (lower W/1 diets) or (lower W/1 dietary) or (higher W/1 enteral) or (higher W/1 nutrition*) or (higher W/1 feeding*) or (higher W/1 energy) or (higher W/1 caloric) or (higher W/1 calorie*) or (higher W/1 diet) or (higher W/1 diets) or (higher W/1 dietary) or (full W/1 enteral) or (full W/1 nutrition*) or (full W/1 feeding*) or (full W/1 energy) or (full W/1 caloric) or (full W/1 calorie*) or (full W/1 diet) or (full W/1 diets) or (full W/1 dietary))
7. 3 and 4 and 5 and 6
8. TITLE-ABS-KEY((randomized W/2 study) or (randomized W/2 studies) or (randomized W/2 trial) or (randomized W/2 trials))
9. 7 and 8
10. PMID(0*) OR PMID(1*) OR PMID(2*) OR PMID(3*) OR PMID(4*) OR PMID(5*) OR PMID(6*) OR PMID(7*) OR PMID(8*) OR PMID(9*)
11. 9 and not 10
12. DOCTYPE(le) OR DOCTYPE(ed) OR DOCTYPE(bk) OR DOCTYPE(er) OR DOCTYPE(no) OR DOCTYPE(sh)
13. 11 and not 12
